# Roles of ESCRT-III polymers in cell division across the tree of life

**DOI:** 10.1016/j.ceb.2023.102274

**Published:** 2023-11-08

**Authors:** Jeremy Graham Carlton, Buzz Baum

**Affiliations:** 1 Comprehensive Cancer Centre, School of Cancer & Pharmaceutical Sciences, Faculty of Life Sciences & Medicine, King’s College London, Guy’s Hospital, London, SE1 1UL, UK; 2 Organelle Dynamics Laboratory, The Francis Crick Institute, 1 Midland Road, London, NW1 1AT, UK; 3 Medical Research Council Laboratory of Molecular Biology, Francis Crick Avenue, Cambridge, CB2 0QH, UK

## Abstract

Every cell becomes two through a carefully orchestrated process of division. Prior to division, contractile machinery must first be assembled at the cell midzone to ensure that the cut, when it is made, bisects the two separated copies of the genetic material. Second, this contractile machinery must be dynamically tethered to the limiting plasma membrane so as to bring the membrane with it as it constricts. Finally, the connecting membrane must be severed to generate two physically separate daughter cells. In several organisms across the tree of life, Endosomal Sorting Complex Required for Transport (ESCRT)-III family proteins aid cell division by forming composite polymers that function together with the Vps4 AAA-ATPase to constrict and cut the membrane tube connecting nascent daughter cells from the inside. In this review, we discuss unique features of ESCRT-III that enable it to play this role in division in many archaea and eukaryotes.

## Cell division

Cell division is fundamental for all life. In unicellular organisms, cell division is the process by which one cell gives rise to two daughter cells to ensure the propagation of the lineage. In multicellular animals, consecutive rounds of cell division generate all the cells required for growth, development, homeostasis and regeneration [1]. In an adult human, millions of divisions occur every second to counter-balance cell loss [2], the majority in blood and intestinal lineages. Cell division is also one of the most complex tasks any cell performs since it requires the precise partitioning of all the component parts of a cell into two daughter cells within a limited time-window. Cell division must occur with high fidelity to ensure accurate segregation of the duplicated genetic material and the fair physical segregation of both cytoplasmic contents and the bounding cell membrane into the new daughter cells. Failures in cell division can lead to cell death and, in the case of multicellular organisms, to the mis-segregation of genetic and cytoplasmic material, which can contribute to cancers and to developmental defects.

### Physics of division

The physical process of division relies on force generation. This is evident in reconstituted systems that mimic the process of division inside synthetic membrane vesicles. In such systems, membrane constriction and separation are promoted via changes in cytoskeletal filament organisation [3], by physical deformation from the outside, via line tension between lipid domains [4], and/or by changes in osmotic pressure that reduce membrane tension to facilitate membrane remodelling [5]. Similarly, in cells, different forces and biophysical factors act together to accomplish division. For example, while membrane constriction in human cells is driven by actomyosin network contraction, these cells also lose volume at around the time of division, likely reducing tension in the membrane [6] as an aid to membrane fission [7]. At the same time, specific lipids accumulate at the site of membrane fission [8], altering membrane biophysics and the membrane association of cytoskeletal polymers to aid division.

Dividing a cell in two also requires energy in the form of Nucleoside triphosphate (NTP) hydrolysis, which is harnessed by a small set of conserved dynamic protein polymers as they perform mechanical work to help generate daughter cells through the process of cytokinesis [9]. The mechanism by which cytoskeletal elements contribute to cytokinesis depends on the system. Thus, in cells that have an external rigid wall and high internal turgor pressure, cytoskeletal polymers that underlie the membrane guide local cell wall synthesis to drive furrow ingression. In this case, it is external cell wall deposition rather than cytoskeletal remodelling that provides the force required to cut cells into two. This is true for fission yeast cells, where organised deposition of the septum depends on the actomyosin ring [10–12], and for many bacteria, where wall synthesis depends on a tubulin-like polymer, FtsZ, often working together with actin-like filaments [13–15]. By contrast, in cells with a soft boundary, like animal cells, *Dictyostelium* [16] and most archaea [17], the forces required for division are generated by cytoskeletal polymers themselves as they pull on the limiting membrane from the inside [18,19]. These forces are balanced by internal resistance, including turgor pressure, and by contacts with neighbouring cells and the extracellular matrix [20,21].

### Regulation of the final stages of cell division

Physically dividing a micron-sized cell into two requires precise regulation. The process cannot begin until the entire set of cellular structures has been duplicated, and until cells have entered a state of readiness. Thus, division in most proliferating animal cells does not begin until cells have first undergone mitotic rounding [22], which likely facilitates fair segregation of material during division [23]. Division must also occur along precisely the right plane. Thus, in animal cells, a signal sent from the region of microtubule overlap at the centre of the anaphase spindle sets up the contractile cortical actomyosin ring and triggers cytokinesis [24]. This actomyosin ring then narrows until the two daughter cells are connected by a membrane bridge only a few microns in diameter that contains the bundled remains of the spindle. Finally, this thin intercellular bridge is cut to yield two physically separated daughter cells in a process called abscission [25]. Abscission typically occurs in the following interphase, often in G1 [26], but can also be delayed in a number of organisms to produce interlinked cysts of daughter cells [27–30]. As this description makes clear, cytokinesis relies on the successful completion of a number of mechanistically distinct steps [27]. Whilst the same machinery could, in theory, drive both ring constriction and membrane scission, in most cases these processes have been shown to be mechanistically distinct. In bacteria, for example, the FtsZ ring driving bacterial cytokinesis is disassembled prior to the completion of cell division [31], implying that FtsZ does not catalyse the final act of membrane scission, even though it has been reported to cut membranes in a reconstituted system [32]. Indeed, whilst FtsZ forms division rings that constrict around mitochondria in red algae, a Dynamin homologue is likely required to complete mitochondrial division from the cytoplasmic side [33], as is the case in plants that lack FtsZ [34]. The same rule holds in human cells, where bundled microtubules and the cortical actin involved in actomyosin ring closure must be disassembled before cells are capable of undergoing abscission [25,35]. The involvement of mechanistically distinct steps in the process of division in all these systems likely reflects differences in the physical requirements of membrane reshaping (constriction) and membrane scission. There is an exception to this rule though. Endosomal Sorting Complex Required for Transport (ESCRT)-III polymers can catalyse both membrane constriction and scission. This remarkable and widely conserved cytoskeletal system is the subject of this review.

## What is ESCRT-III?

ESCRT-III proteins are members of an evolutionarily ancient family of filament forming proteins that are present across all domains of life. ESCRT proteins were originally identified in a series of screens [36–40] as genes that when mutated disrupt the sorting of proteins into the lumen of the yeast vacuole. In the early 2000s, the Emr lab demonstrated that the products of these genes assemble into three separate hetero-oligomeric complexes which they named the ESCRT —I, —II and —III [41,42]. In budding yeast, the ESCRT machinery functions to sort cargo into the lumen of the vacuole. Here, ESCRT-I and ESCRT-II act together to corral ubiquitin-modified transmembrane proteins into infoldings of the endosomal membrane [43]. Ubiquitinated cargo binds ESCRT-I, and this complex forms polymers, together with ESCRT-II, that help template the formation of ESCRT-III filaments [44]. Changes in the structure of membrane-bound ESCRT-III polymers [45,46] then re-shape and sever the limiting endosomal membrane to release intralumenal vesicles containing ubiquitinated transmembrane cargo into the endosomal lumen for later degradation in the lysosome or vacuolar lumen [43].

To achieve this feat of membrane remodelling, ESCRT-III proteins act inside the lumen of cytosol-filled membrane tubes that connect membranes that need to be separated. They sever this connection via performing reverse-topology membrane fission, i.e. cutting the membrane tube from within. This is opposite to the normal-topology of membrane fission performed by protein assemblies like Clathrin, COPI or COPII, which catalyse the formation of membrane-bound vesicles [47] that bud into the cytoplasm. The act of cutting a cytosol-filled membrane tube from the inside also distinguishes ESCRT-III from Dynamin and actin filaments which induce the scission of membrane tubes by squeezing around their outer cytosolic sides [48]. It is this unique ability that makes ESCRT-III critical for the maintenance of the dynamic intracellular architecture of all eukaryotic cells. Thus, ESCRT-III homologues catalyse a wide variety of cellular processes that require reverse topology membrane fission. ESCRT-III proteins function in the budding and release of extracellular vesicles [49–51] and enveloped retroviruses, such as HIV-1 [52–55]; in the repair of membrane organelles [56–58]; the closure and sealing of autophagosomes [59]; the sealing of holes in the nuclear envelope, at mitotic exit [60,61] and following nuclear envelope rupture [62,63]; and induce the structural remodelling of neural processes [64]. As we will see, ESCRT-III homologues also play an important role in late stages of division in mammalian cells, a process that requires a topologically equivalent membrane fission event during abscission — the final stage of cytokinesis [65,66].

## Key features of ESCRT-III polymers

How is ESCRT-III able to deform and cut membrane tubes from within? Like many other cytoskeletal elements, ESCRT-III proteins assemble into dynamic polymers to perform their cellular functions. However, there are a several features that distinguish ESCRT-III polymers from the more familiar cytomotive filaments, microtubules and actin [9]. These features of ESCRT-III play a key role in the ability of its polymers to remodel and cut membrane tubes from the inside.

First, while ESCRT-III proteins form flexible homopolymers, each of which has a slightly different preferred curvature, these come together as part of composite filaments, whose nature changes as subsets of ESCRT-III monomers are added and/or removed. This is possible because from archaea to eukaryota, all cells co-express multiple different ESCRT-III isoforms. The coming and going of individual ESCRT-III proteins drives sequential changes in the structure of the composite polymer [67–69], which are thought to guide the membrane through a series of different states from deformation, to construction and fission. Importantly, a similar type of behaviour has been observed in reconstituted systems. As an example of this, the growth of spirals of Snf7, the major filament forming ESCRT-III subunit in yeast, is limited by the addition of Vps2 and Vps24 subunits, which co-polymerise to form lateral filament bundles [70]. At the same time, spirals of an activated mutant of Snf7 can be transformed into 3-dimensional helices through the addition of Vps2 and Vps24 [71], which may then buckle the underlying membrane as a prelude to scission. In an extension of these analyses, Roux et al. found that when different ESCRT-III monomers were pre-mixed with the ESCRT-associated AAA–ATPase, Vps4, and added to membranes *in vitro*, the polymers formed underwent changes in composition as different ESCRT-III monomers were incorporated and removed in sequence [67]. Because the changes in subunit incorporation occurred in a fixed order, this has the potential to ensure that membrane remodelling proceeds to completion [67,69]. Several factors likely contribute to the precise ordering of ESCRT-III monomer recruitment and release in this case. These include intrinsic differences in the biochemistry of polymer assembly (the nucleation rate, elongation rate and lateral filament—filament interactions), differences in preferred curvature that allow each ESCRT-III polymer in a series to bind increasingly deformed membranes, and differences in the affinity of different ESCRT-III subunits in a polymer for Vps4, the AAA-ATPase that catalyses ESCRT-III polymer disassembly (see below). Importantly, since sequential changes in filament composition translate into changes in polymer structure, the diameter of composite filaments can narrow over time to constrict the membrane tube to which it is bound. The idea that sequential changes in ESCRT-III copolymer structure might drive membrane remodelling in this way aligns well with theory and coarse-grained molecular dynamics simulations [19,69,72,73]. Such models show how shifts in filament geometry and in the orientation of the polymer’s membrane binding surfaces can drive the transition from flat spirals to helices that disassemble to induce fission. They also provide a theoretical framework for understanding how the ESCRT-III filament operates to shape membranes and make predictions that can be tested experimentally [19].

ESCRT-III polymers also harness energy in a unique way. Unlike actin or tubulin, ESCRT-III monomers neither bind nor hydrolyse nucleotides. Instead, the energy they require to do work is supplied *in-trans* via the action of the AAA-ATPase, Vps4 [45], which extracts individual monomers from pre-formed filaments in an order that is determined in part by the affinity of Vps4’s Microtubule Interaction and Trafficking (MIT) domain for sequence motifs in the C-terminus of each ESCRT-III monomer [74]. The activity of Vps4 depends on its ATPase domain, which form a staggered hexameric ring around C-terminal regions of ESCRT-III monomers. Through ATPase-driven monomer dissociation and rebinding, Vps4 extracts proteins from the polymer through the hexamer’s central pore [75]. This activity of Vps4 is thought to drive subunit exchange within the ESCRT-III filament, leading to sequential changes in composite filament structure that direct membrane remodelling [67,70].

In addition, whereas actin and tubulin form linear polymers, the filaments formed by individual ESCRT-III monomers are inherently curved, forming flat spirals and helices [71,76–78]. ESCRT-III filaments can also form hetero-polymers (e.g. Vps2/Vps24 or CHMP2/CHMP3) and composite filaments generated via the lateral association of different homopolymers [79], which may widen the range of preferred geometries that the composite filament can access. Because each filament has a preferred geometry, filaments recruited into composite polymers that are forced to deviate from their ideal shape can store mechanical energy that can be later deployed to remodel the membrane [72,77,80] once neighbouring filaments that constrain it have been removed by Vps4.

Lastly, unlike actin and tubulin, ESCRT-III filaments bind and polymerise on membranes, enabling alterations in filament architecture to be transferred directly to the overlying membrane. ESCRT-III polymers interact with the membrane in part through electrostatic forces [54,81]. In addition, the recent CryoEM structures of the CHMP2A/CHMP3 and CHMP1B/IST1 helical polymers reveal the presence of hydrophobic residues that engage in an intimate interaction with the hydrophobic core of lipids in the membrane’s outer leaflet [82,83]. This partial insertion of ESCRT-III polymers into the membrane may be important to deform the membrane as the filament narrows and to destabilize the membrane so that fission can occur. In addition, ESCRT-III associated proteins, like ALIX [84], help ESCRT-III polymers to associate with membranes, in this case by forming a complex with Syntenin and its transmembrane partner Syndecan-4, to aid plasma membrane tethering during cytokinesis [85].

### Reconciling ESCRT-IIII filament structure and function

Although ESCRT-III polymers can take on a wide array of structures, as evidenced by the wide array of ESCRT-III polymers visualised by X-ray crystallography and cryoEM, in nearly every case, neighbouring monomers along the length of a proto-filament bind to one another via a common interface linking helix 5 to helices 1 and 2 in adjacent subunits [83,86–88]. Despite sharing this common mode of interaction, flex at several hinge regions in the monomer enable filaments to take on a range of forms. In addition, adjacent filaments can associate with one another via lateral electrostatic interactions, for example, in the multi-stranded assembly of CHMP2A and CHMP3, or between separate Snf7 and Vps24 filaments [89,90], a feature of the system that may allow adjacent filaments in a multistranded polymer to slide over one another without much resistance. This may be important to allow macro-scale reorganisation of composite filament architecture during membrane remodelling, e.g. enabling the closure of a molecular garrotte [91]. At the same time, hydrophobic interactions between C-terminal regions and the helical hairpin cores of adjacent protofilaments may restrict such sliding, providing a possible way for accessory proteins that bind these C-terminal helices to control filament remodelling.

A major problem with the search for the structural sources of flexibility in ESCRT-III polymers is the fact that most CryoEM structures are solved by averaging large numbers of images representing uniform classes of structures. Nevertheless, hints of conformational flexibility can be gleaned from the available structural datasets. For example, an analysis of different multiple class averages of membrane-coated CHMP2A and CHMP3 co-polymers has revealed how the removal of a single monomeric unit can reduce the diameter of the co-polymer [83], suggesting a mechanism by which Vps4-catalysed subunit extraction may contribute to neck tightening and scission [92]. Even more strikingly, bacterial ESCRT—III—like homopolymers form caps with different symmetries. Individual monomers in each of these structures take up a wide range of conformations to form stacked rings of different diameters that contain different numbers of subunits [86,87]. While static, these structures highlight the ability of ESCRT-III monomers to take on different conformations when polymerising into filaments.

An additional problem in reconciling structural studies with the cell biological data is that, with some exceptions (e.g. work on CHMP2/CHMP3 co-polymers [83] in which lipids were nucleated on top of pre-formed filaments), most structural studies have examined polymers bound to membrane tubes from the outside. While such structures may, in some cases, play functionally important roles in cells (e.g. Vipp1 [87]), this bias reflects the simple fact that it is much easier to access membranes from the outside when working *in vitro*. Nevertheless, the ability of polymers that typically bind and cut membrane tubes from the inside *in vivo* to form well-defined structures on the outside of membrane tubes *in vitro*, emphasizes the inherent flexibility of ESCRT-III polymers. Moreover, it is possible that large-scale changes in the structure of dynamic ESCRT-III polymers accessed in these structural studies could be functionally important in cells, e.g. enabling the formation of cones that induce the membrane to narrow towards a neck [92,93], or by flipping the orientation of membrane binding sites [72] to induce abscission.

## The evolution of ESCRT-III

Given the focus on the roles of ESCRT-III proteins in endomembrane trafficking in eukaryotic cells, it was a great surprise when homologues of ESCRT-III were discovered in archaea [94,95], which for the most part lack internal membranes. Since then, ESCRT-III homologues have been found across the tree of life (Figure 1). These phylogenetic analyses suggest that the last universal common ancestor of all cells (LUCA) likely possessed homologues of one or more ESCRT-III proteins, which gave rise to bacterial and archaeal ESCRT-III proteins. One or more archaeal homologues were then inherited by the last eukaryotic common ancestor during the emergence of eukaryotes.

This realisation has profound implications for our understanding of ESCRT-III function. Bacteria likely inherited a single homologue of ESCRT-III from LUCA, variously called PspA/Vipp1/IMO30/LiaH, which forms polymers in many bacteria that function in membrane remodelling and repair [86,87,96,97]. Interestingly, bacterial genomes tend to encode a single copy of this protein. While these PspA homologues sometimes work together with an ATPase (PspF), bacteria lack a clear homologue of the Vps4 AAA-ATPase [86]. This raises the possibility that PspA polymers function by forming a physical platform that stabilises membranes in a manner that is regulated by non-Vps4 AAA-ATPases and/or by the direct binding and hydrolysis of NTPs, as suggested by a structural analysis of Vipp1 [87]. By contrast, most archaea possess multiple ESCRT-III homologues, which seem to form co-polymers that act together with Vps4 to remodel archaeal membranes to catalyse the formation of extracellular vesicles during viral release and cell division [98–100]. Interestingly, while very few archaea possess a single ESCRT-III homologue, many possess just two [86]. This includes most Asgard archaea, which possess two versions of these two proteins that we call ESCRT-IIIA and ESCRT-IIIB. During the process of eukaryogenesis, homologues of ESCRT-IIIA and ESCRT-IIIB appear to have given rise to the two major families of eukaryotic ESCRT-III proteins, named CHMP1-3 and CHMP4-7 families respectively [99,101,102]. The later divergence of these ESCRT-III family proteins in eukaryotes (Figure 1) likely widened the range of spatial scales over which they can act, reduced the energetic barrier to membrane remodelling [69] and enabled specific ESCRT-III isoforms to adapt to specific eukaryotic contexts, e.g. nuclear envelope sealing [103].

## A comparison between ESCRT–III-dependent abscission in human cells and ESCRT–III-mediated division in archaea

While ESCRT-III proteins have diverse functions across the tree of life, by focussing on their function in similar cell biological process in distantly related organisms, it is possible to identify conserved features of ESCRT-III function and common physical principles involved in ESCRT-III-dependent abscission (see Figure 2). Cell division is a great case in point since during this universal process one can observe how ESCRT-III polymer assembly and disassembly are regulated to ensure that scission occurs, with precision, at the right time and place. Thus, in the next section, we discuss what is known about the likely mechanism of ESCRT-III-mediated abscission in eukaryotes and archaea — organisms that likely shared a common ancestor approximately two billion years ago.

### ESCRT-III-dependent abscission in human cells

While there are species-specific differences in the importance of ESCRT-III for division across eukaryotes, in many of these systems, ESCRT-III aids the timely membrane remodelling required to complete the division process. In most cases, the process of ESCRT—III—mediated abscission is triggered by mitotic exit, necessitating a regulatory link between ESCRT-III function and mitosis (but see the study by Chaigne et al. [104]).

The role of ESCRT-III polymers in division is best understood in human cells (Figure 2). At the onset of anaphase, as human cells partition their component parts into two, divide, and begin re-establish their interphase form, ESCRT-III polymers first seal and repair holes in the rapidly reforming nuclear envelope [60,61]. Then, as cells enter telophase and the cleavage furrow contracts around the remnants of the tubulin-based mitotic spindle, a midbody is formed at the cell centre (Figures 2a and 2c). This midbody is generated by PRC1, Centralspindlin, CEP55 and the Chromosome Passenger Complex, which are recruited by antiparallel microtubules at the centre of the anaphase spindle [24,26] (Figures 2a and 2c). The enormous, relatively insoluble protein complex that remains, also known as the Flemming body, binds tightly to the overlying plasma membrane [85]. Being close to a micron in diameter, this is both hard to disassemble and is stable enough to be isolated for proteomic analyses [84,105]. As a result, rather than serving as the site of abscission, the midbody is used by cells as a platform to recruit the abscission machinery. Scission then occurs some distance away, in a region where the diameter of the membrane tube connecting daughter cells is thinner. Abscission is initiated when midbody components (including CEP55 [65,66] and Centralspindlin [106]), recruit ESCRT-I, ESCRT-II and/or adaptor proteins such as ALIX [107–109]. These upstream factors recruit ESCRT-III proteins, which assemble into helical polymers that constrict the membrane at some distance from the Flemming Body on either one or on both sides (Figure 2a). Once cortical actin and central spindle microtubules at one of these sites have been disassembled, as the result of changes to the local lipid composition, the activity of actin-oxidases and Spastin-dependent disassembly of remnant spindle microtubules [35,110,111], the membrane is able to undergo ESCRT—III—dependent membrane scission.

In catalysing this process, ESCRT-III proteins are thought form composite helical polymers that constrict and cut the intercellular bridge. Snapshots of ESCRT-III at the site of abscission include multiple components, and appear to form helical cones that could help narrow the neck until it can serve as a site for abscission [110,112,113] (Figure 2a). This occurs with the help of Vps4. However, while Vps4 has been proposed to be required for polymer assembly as well as filament remodelling [70], it remains unclear precisely how it contributes to the process.

Abscission is also subject to complex regulation by both biochemical signalling and mechanics. For example, mammalian cells possess different isoforms of CHMP4, which are subject to regulation via Aurora B-mediated check-point control [114,115] and, in flies, post-translational modification of ESCRT-III units by ubiquitination has been shown to control abscission progression [27]. Furthermore, membrane tension appears to limit ESCRT—III—dependent abscission in mammalian cell culture [7], in a process that may be influenced by local caveolae [116] and by ion flow through Piezo1, a mechanosensitive calcium channel that controls the recruitment of ESCRT-III to the midbody [117].

### ESCRT–III–dependent abscission in TACK archaea

Within the archaea, ESCRT-III mediated division is best understood in *Sulfolobales*. These members of the Thaumarchaeota, Aigarchaeota, Crenarchaeota, and Korarchaeota (TACK) archaea resemble eukaryotes in having an ordered cell division cycle [118], which are associated with periodic transcriptional waves, and lack FtsZ [119]. By identifying the genes that are expressed in a wave of transcription that precedes cell division in *Sulfolobus acidocaldarius* [94,118], Bernander et al. discovered a set of “**C**ell **d**i**v**ision” genes they termed CdvA, CdvB and CdvC, that are co-located in the genome. While CdvA does not have close homologues outside of TACK archaea (despite possessing a PRC domain that is found in bacterial [120,121] and archaeal [122] division proteins), CdvB encodes an ESCRT-III homologue and CdvC is a homologue of Vps4 [94,118]. This discovery suggested that, like their eukaryotic counterparts, CdvB and CdvC proteins function in cell division [65]. In support of this idea, CdvA and CdvB form rings in the middle of cells prior to division [94,118,123,124] (Figure 2b).

Although the detailed genetic analysis of this Cdv cluster is complicated by the fact that the genes for CdvA, B and C are all essential for viability [124,125], the interaction between CdvA and CdvB proteins (mediated via complementation of the broken winged helix domain in CdvB by CdvA) has been shown to be important for division, along with the AAA-ATPase activity of the Vps4 homologue, CdvC [79,118,124]. In parallel, biochemical and structural studies have revealed that both CdvA and CdvB form polymers that can associate with membranes [120,121]. It was subsequently discovered that *Sulfolobales* genomes encode several other ESCRT-III homologues, termed CdvB1, CdvB2 and CdvB3 [124,126]. Interestingly, while CdvB1 and B2 are not co-located with CdvABC in the genome, they form part of the same transcriptional wave, form rings at division, and play important roles in the act of division itself in *Sulfolobales* [68,79,124,127]. Despite individual species differences, high level expression of CdvB1 or CdvB2 variants that lack the C-terminal CdvC/Vps4-binding region, which prevent polymer disassembly [79,126], impair division in *Sulfolobales*. In *Sulfolobus islandicus*, this leads to chains of connected cells, implying a failure in abscission [126]. Moreover, in *S. acidocaldarius*, C-terminally truncated CdvB1 accelerates cell constriction, while abscission is compromised by C-terminally truncated CdvB2, suggesting functional divergence in the roles of polymers formed from these two ESCRT-III subunits [79]. While CdvB1 and CdvB2 appear essential in *S. islandicus* [126], in S. *acidocaldarius*, where these deletions are viable, the deletion of CdvB1 compromises cell constriction, whereas the deletion of CdvB2 leads to misplacement of the division site and to severe defects in abscission [127]. By contrast, CdvB3, which is not part of the pre-division transcriptional wave, appears to encode a small protein with a core 5 alpha-helical ESCRT-III fold but no C-terminal extension. CdvB3 is important for budding of viruses from Archaea [126,128], suggesting that the co-option of ESCRT-III to facilitate viral egress is a similarly ancient event. There is less evidence that CdvB3 plays a direct role in division (although see the study by Yang et al. [124]).

Building on these genetic data, a detailed set of cell biological studies has helped to define the sequence of events that underlie archaeal division using a combination of live cell imaging and an analysis of populations of synchronous cell cultures fixed at various times after release from a cell cycle arrest. First, a CdvA ring is assembled at the midzone [120] via a process that has not yet understood. This CdvA ring recruits a ring of the ESCRT-III subunit, CdvB, which in turn recruits ESCRT-III homologues CdvB1 [129] and CdvB2, which assemble into adjacent rings on the membrane. Cells with composite ESCRT-III rings at their centre are ready to divide. Then, an as yet unknown trigger causes CdvB to be extracted from the ring, via the action of CdvC [79]. CdvB is then degraded by the proteosome [68]. The sudden loss of CdvB from the composite ring triggers rapid cell constriction (Figures 2b and 2c), perhaps by enabling CdvB1 and CdvB2 polymers to adopt their preferred curvatures. In this process, CdvB1, which has a broader localisation, appears to control the rate of membrane constriction since this is lower in the mutant and is enhanced by the overexpression of wild-type CdvB1 or a truncated variant of the protein [79]. CdvB2, which is localised to the centre of furrow, then appears to execute the final cut. Importantly, scission also likely requires CdvC-dependent disassembly of CdvB2 since a truncated CdvB2 that cannot be extracted from the division ring arrests cells with thin division bridges [79,126]. Thus, although CdvC, the Vps4-like AAA-ATPase, does not appear to be required for timely ring assembly in *Sulfolobus* [79], it is required throughout the disassembly process to induce the transition in states that drives division —just as has been suggested for its eukaryotic counterpart. In this system, CdvC/Vps4 also likely helps to dictate the order of ESCRT-III polymer disassembly, through differential affinity of for sequence motifs within the tails of the different ESCRT-III proteins, so that high affinity substrates are removed first [95]. Finally, in way that is not yet understood, this process of division must be coordinated with DNA segregation [127] so that the constriction of the midzone leads to the formation of two daughter cells each of which carries a single copy of the genome.

## Conclusion

As we have seen in this review, a conserved membrane remodelling complex, ESCRT-III, plays a critical role in driving division in human cells and in *Sulfolobus* cells [50,79], organisms that likely shared a common ancestor more than two billion years ago. While this is remarkable, it is important to point out that, although ESCRT-III plays a common role in division in these two very distantly related systems, this is likely a consequence of convergent evolution, i.e. as organisms on different branches of the tree of life independently evolved to use shared machinery to perform the same process. Evidence for this being the case comes from the fact that Asgard archaea, which lie between TACK archaea and Eukaryotes on the phylogenetic tree [101,130] (Figure 1), possess a classical FtsZ; a protein that is usually associated with division in archaea [119]. Furthermore, in the genomes of Asgard archaea, ESCRT-III homologues and Vps4 tend to be co-located alongside ubiquitin and ubiquitin-related machinery that includes ESCRT-I and II homologues implying a role for Asgard ESCRT-III in membrane protein quality control [99].

Nevertheless, when compared, the archaeal and human systems can help reveal generic principles of ESCRT-III function that have remained unchanged over long periods of evolution (Figure 2c). In addition, they can shed light on the common mechanisms of control that regulate ESCRT-III-dependent membrane scission in space and time and that coordinate this process with the cell division cycle and genome segregation [114,127,131] (Figure 2c). As examples of this, in both *Sulfolobus* and human cells, abscission occurs at a site determined by the preassembly of other machinery (the midbody or CdvA, respectively). In both cases, ESCRT-III polymers cut a membrane tube that is about 1 micron in width. Furthermore, in both *Sulfolobus* and human cells, scission is driven by Vps4-dependent remodelling of ESCRT-III polymers that are composite in nature and include polymers that form helical cones that appear to draw the membrane to a point.

Extrapolating from these findings, one might expect to see similar features play out during division and other ESCRT—III-mediated processes across the tree of life. For example, while as yet there is little evidence for PspA and other bacterial ESCRT-III homologues assisting in abscission, FtsZ does not appear to execute the final step [31]. This suggests the possibility that ESCRT-III homologues may play a role in this process in some bacteria. Thus, while there is much we do not yet understand about the mechanism by which ESCRT-III polymers deform and cut membranes, continuing the study of ESCRT-III homologues in the division of cells from across the tree of life promises to reveal many more of ESCRT-III’s secrets.

## Figures and Tables

**Figure:**
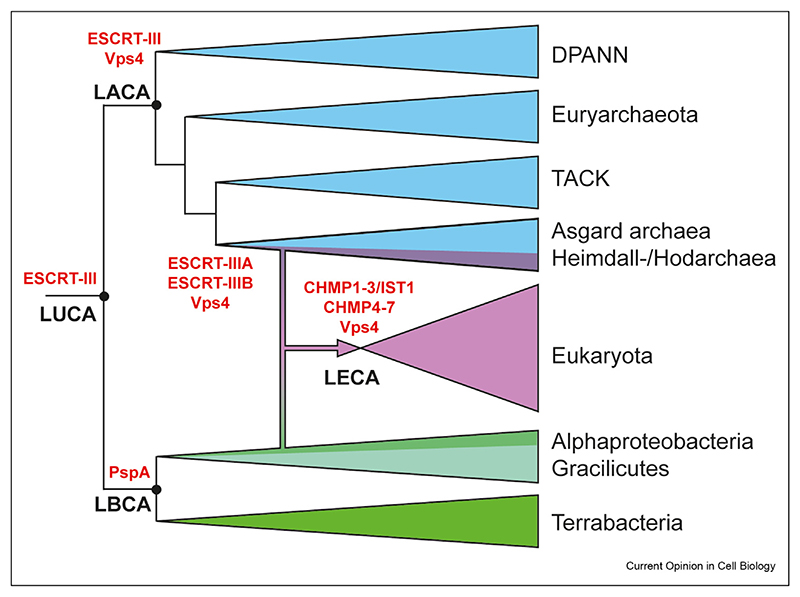
The evolution of ESCRT-III Figure shows the likely evolution of ESCRT-III proteins [100,102] during the history of life on earth based on recent phylogenetic data [101]. ESCRT-III proteins that form filaments that interact with membranes were likely present in the ancestral cell that gave rise to all the domains of life (LUCA). This ancestral form of ESCRT-III likely gave rise to both archaeal and bacterial versions, which we respectively call ESCRT-III (inherited by the last common ancestor of all archaea, LACA) and PspA (inherited by the last common ancestor of all bacteria, LBCA) [86]. Importantly, while archaea tend to have multiple copies of ESCRT-III proteins that form composite polymers remodelled by Vps4, in the genomes of bacteria, ESCRT-III proteins are often found as single proteins, and may not function together with an AAA ATPase, equivalent to Vps4. The last common ancestor of all Asgard archaea likely inherited two versions of the protein, which we term ESCRT-IIIA and ESCRT-IIIB, that work together. These were then inherited by the cells in the lineage that gave rise to eukaryotes [130] through a merger with an alphaproteobacterial partner that gave rise to mitochondria. During the evolution of eukaryotes, these two versions diversified to give rise to a set of eight ESCRT-III proteins that are related to both ESCRT-IIIA and ESCRT-IIIB, which were inherited by LECA — the last common ancestor of all eukaryotes.

**Figure:**
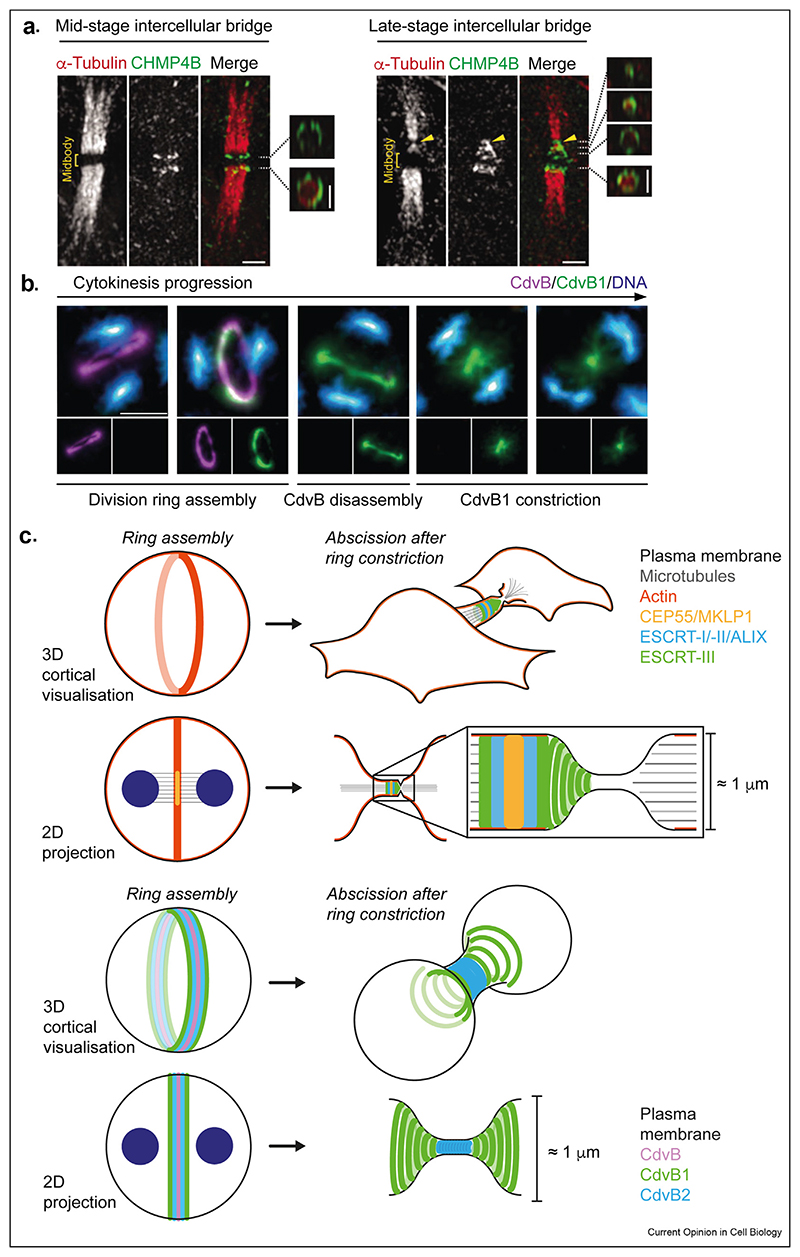
Principles of abscission a. Image (from the study by Guizetti et al. [110]) shows structured Illumination Microscopy of the midbody of HeLa cells stably expressing CHMP4B-EGFP expressed with its endogenous regulatory sequences from a bacterial artificial chromosome and an EGFP-containing Localisation And Purification (LAP) tag [110], and stained for endogenous Tubulin, at mid- and late-stages of cytokinesis. Scale bar is 1 μm. Boxed regions show cross sections and the relative position of ESCRT-III and Tubulin at the indicated positions along the midbody. In early- and mid-stages of abscission, ESCRT-III assembles as large diameter rings on each side of the Flemming body. As abscission progresses, ESCRT–III–dependent cortical constriction narrows the midbody and ESCRT-III polymers extend to the abscission site (yellow arrowhead). Midbody microtubules are disassembled in the ESCRT–III-positive zone, culminating in an absence of microtubules from the abscission site to permit membrane fission and separation of daughter cells. Note, in this figure, the term ‘midbody’ has been used to refer to the central dark zone in which Tubulin epitopes are obscured by the protein-dense Flemming body. In this review, we refer to this region as the ‘Flemming Body’ and the entire inter-cellular bridge as the midbody. Images reproduced and modified with permission from the study by Guizetti et al. [110]. b. Super-Resolution Radial Fluctuation microscopy of dividing *Sulfolobus acidocaldarius* cells stained with antisera raised against CdvB and CdvB1, and with DAPI. In *S. acidocaldarius* cells at early stages of division with segregated nucleoids and large division rings, CdvB and CdvB1 are colocalised (note that CdvB2 and the cell surface are not shown here). Ring constriction is triggered by Vps4-mediated extraction of CdvB from the ring [79] followed by rapid proteasome-mediated degradation [68]. This leaves composite CdvB1 and CdvB2 rings, which constrict to generate a bridge, with CdvB1 at the flanks and CdvB2 at the midzone. The disassembly of CdvB2 then likely induces scission. Images reproduced from the study by Tarrason Risa et al. [68]. c. A model comparing abscission in *Sulfolobus* and human cells. In both systems, a contractile ring assembles at the midzone. In human cells, and in other eukaryotes, this is formed from filaments of actin with contractility supplied by the motor protein myosin. Central spindlin components and CEP55 assemble at midzone microtubules. Cartoon depicted at anaphase-A where chromosome segregation has initiated, but furrow ingression has not yet begun. In *Sulfolobus,* this ring comprises CdvB and straddles the separated nucleoids. In human cells, a midbody is generated as the contractile ring ingresses and the spindle microtubules are bundled. As the midbody forms, additional ESCRT proteins assemble alongside the centrally placed Central spindlin and CEP55. In human cells, this includes ESCRT-I, ESCRT-II, ESCRT-associated proteins such as ALIX and ESCRT-III. In *Sulfolobus*, constriction is achieved through recruitment of ESCRT-III components CdvB1 and CdvB2 to the CdvB ring at the cell centre. Following the extraction of CdvB by Vps4, CdvB1 and CdvB2 are free to constrict, narrowing the bridge between daughter cells. In human cells, a secondary ingression is thought to be form via polymerisation of a composite ESCRT-III polymer, the removal of cortical actin and the disassembly of midbody microtubules, bringing membranes into close opposition for fission. In *Sulfolobus,* while both CdvB1 and CdvB2 narrow the bridge between daughter cells, the disassembly of centrally positioned CdvB2 by Vps4 likely induces membrane scission.

## Data Availability

No data were used for the research described in the article.

## Box

For complete overview of the section, please refer the article collection - Membrane Trafficking 2023
